# A computational framework for ptychographic reconstructions

**DOI:** 10.1098/rspa.2016.0640

**Published:** 2016-12

**Authors:** B. Enders, P. Thibault

**Affiliations:** 1 Department of Physics & Institute of Medical Engineering, Technical University of Munich, 85747 Garching, Germany; 2 Advanced Light Source, Lawrence Berkeley National Laboratory, Berkeley 94720, CA, USA; 3 School of Physics and Astronomy, University of Southampton, Southampton SO17 1BJ, , UK

**Keywords:** X-ray, microscopy, ptychography, diffraction, Python, phase retrieval

## Abstract

Ptychography is now a well-established X-ray microscopy tool for synchrotron end-stations equipped with a scanning stage and a pixelated detector. Ptychographic phasing algorithms use information from coherent diffraction to deliver quantitative images of the specimen at a resolution higher than the scanning resolution. These algorithms have traditionally been implemented in software on a per-instrument basis in various degrees of user-friendliness and sophistication. Here, we present Ptypy, a ptychography software written with the intention to serve as a framework across the diverse sets of available instruments and usage cases. A distinctive feature of the software is its formalism, which provides a convenient abstraction of the physical model, thus allowing for concise algorithmic implementations and portability across set-up geometries. We give an overview of the supported usage cases, explain the abstraction layer and design principles, and provide a step-by-step guide describing how an algorithm may be realized in a concise and readable manner. The software capabilities are illustrated with reconstructions from visible light and X-ray data.

## 1. Introduction

In the last decade, coherent diffractive imaging (CDI) techniques have evolved as a promising dose-efficient and high-resolution complement to traditional, lens-based, X-ray microscopy [1–3]. Though they vary in their implementation, CDI techniques share the essential characteristic that sample images are taken indirectly, via the collection of diffraction patterns, thus avoiding potential signal degradation caused by image-forming optics. However, this benefit comes at a cost: the image can be recovered only by solving an inverse problem—most often a computationally demanding task.

Among CDI techniques, ptychography has been especially successful at delivering quantitative images of extended specimens at diffraction-limited resolution. Originally developed in the late 1960s for electron microscopy [4], ptychography combines multiple diffraction patterns formed by a finite illumination while it is scanned over an extended specimen. The technique saw an important revival when Faulkner & Rodenburg [5] demonstrated that iterative algorithms used for other types of CDI techniques could be adapted to tackle the inverse problem in ptychography.

An essential feature of ptychography is the imposition of self-consistency for illuminated areas at adjacent scan points, often called the ‘overlap constraint’. It is now known that the resulting redundancy in the acquired data is sufficient not only to recover simultaneously the illumination profile and the specimen image [6,7], but also to overcome experimental limitations spanning from inaccurate scanning positions [8,9] to diffraction data degradation effects [10] such as point-spread-function and air scattering [11] or sample jitter [12]. Hence, unlike single-shot CDI [13,14], ptychography can handle various sources of data degradation by adapting the propagation models and without imposing *a priori* knowledge.

Beyond its far-field diffraction version, ptychography is now applied to other microscopy techniques by including transverse scanning of the sample to generate data redundancy. Ptychographic principles can be transferred to the holographic regime [15,16] or to Bragg geometry [17,18], and have also been mapped to reciprocal space [19]. In addition, the quantitative nature of the technique lends itself naturally to tomographic applications, thus yielding quantitative volumetric information at the nanoscale [20,21].

Reconstruction algorithms form an essential part of ptychographic techniques. The last decade has seen important progress in the way computer algorithms explore the high-dimensional phase space of possible sample transmission and illumination functions in the search for a unique solution. The first occurrence of an iterative algorithm was the *ptychographic iterative engine* (PIE) [5], which addressed the ptychographic reconstruction problem through sequential updates inspired by previous reconstruction approaches [1,22,23]. The technique is still widely used today in one of its updated versions (*ePIE* [24], *3PIE* [25]). Although the PIE family iteratively cycles through the diffraction patterns, other algorithms act in a parallel manner, following a generalized projection formalism. Notable examples are the *difference map* (DM) [7,26], the *relaxed averaged alternating reflections* (RAAR) algorithm [27] and other similar formulations [28]. Other parallel reconstruction techniques originate from cost–function optimization techniques [6] such as those based on *maximum likelihood (ML) principles* [29].

Reconstruction techniques are still evolving at a rapid pace. A survey of the literature shows that several independent implementations of these algorithms exist, most of them including additional ad hoc capabilities depending on specific experimental conditions [8,12,30,31]. Up to now only a few groups have made their code available to the wider scientific community:

(i) A set of MATLAB scripts implementing the *difference map* and *ePIE* algorithms and developed at the cSAXS beamline of the Swiss Light Source in 2007–2009 [7,20,32] is available for the users of the beamline.
(ii) The Sharp Camera package [33] was developed by the *Center for Applied Mathematics for Energy Research Applications (CAMERA)* and is hosted at http://www.camera.lbl.gov/. It features parallel reconstruction on multiple graphics processor units (GPUs) in far-field geometry using RAAR as the reconstruction algorithm [34]. The package uses the proprietary CUDA parallel computing toolkit for nVIDIA GPUs.
(iii) The software mentioned in [35] was briefly available online but is currently unavailable [36,37]. It provides an implementation of *ePIE* and was implemented for parallel reconstructions on multiple GPUs again for nVIDIA’s CUDA framework only.

The availability of stable reconstruction packages has clear benefits for the development of a technique:

(i) It allows data from different set-ups to be *compared*, and results to be *validated* after *disclosure* of data and reconstruction parameters.
(ii) It furthers the *development of standards*, which is helpful for both a *unified formalism* and *quality control*.
(iii) It lowers the *entry barrier* for new groups resulting in an *expansion of the user base* leading to a *wider acceptance* for the method.

Considering that many high-resolution nano-probe beamlines are planning to support ptychography as a standard, and that ptychographic algorithms have reached a sufficient level of maturity, it has become clear that the community needs broader access to state-of-the-art reconstruction software.

In this paper, we describe PtyPy, an open-source software framework for ptychography written in Python. An essential design feature of PtyPy is the clear separation between representations of physical experiments, the *models*, and the algorithmic implementations to solve the inverse problem, the *engines*. Beyond basic design principles, this paper provides an overview of the supported models and shows how the abstraction from the physical experiment is achieved. An in-depth walk-through of a basic implementation of the *difference map* algorithm demonstrates how such abstraction and the associated programming interface of PtyPy results in concise, readable and intuitive reconstruction engines despite possible model complexity.

## 2. Ptychography

Coherent diffractive imaging is often described as a lensless technique as it lets an image form naturally from coherent propagation (diffraction) contrast, without any optics between the sample and the detector. The contrast mechanism is restrictive because it applies only to highly coherent sources, but it is also inherently dose efficient because no photon exiting the sample is absorbed or scattered by additional optics. Hence, CDI techniques are especially well suited to making good use of the high-brilliance X-rays produced by third- and future fourth-generation synchrotron sources.

Let **x** be a two-dimensional coordinate in the plane transverse to the incident radiation and *ψ*(**x**) be the wavefield just behind the sample, the exit wave. In the model of CDI for a detector in the far field, the intensity measured in the detector plane can be written as the squared modulus of the Fourier transform of the exit wave

2.1 $$I(\text{v})=|F\{\psi (\text{x})\}(\text{v}){|}^{2}={\left|\int_{R^{2}}\psi (\text{x})\cdot e^{-2\pi i\text{v}\text{x}}\;d\text{x}\right|}^{2},$$

where the reciprocal-space coordinate **v** represents spatial frequencies in the real-space image and is related to the detector coordinate **s** by the simple geometric relation

2.2 $$\text{v}=\frac{\text{s}}{\lambda z},$$

with λ the wavelength of the radiation and *z* the sample-to-detector propagation distance. Hence, CDI is an indirect imaging technique, and the final image must be retrieved computationally.

In cases where sufficient holographic information from a pinhole or any other reference [38,39] is encoded in the diffraction signal, the image retrieval may be as straightforward as an inverse Fourier transform with an optional differential operator. However, without a structural reference, the general CDI model (2.1) faces two important difficulties. First, collected data lack the phase part of the propagated exit wave *ψ* as only a time-averaged intensity signal is collected—this is the *phase problem*. Second, even if it is known, the exit wave is itself a composite of incoming illumination *p* and the sample transmission *o* which can be modelled as a product in the simplest approximation (projection) but is in general more complex for a thick sample (see, for example, the supporting online material of [7]).

The difficulty of CDI is further increased by other technical complications. For instance, the high dynamic range in the diffraction signal sometimes extends beyond the limited dynamic range of today’s linear detectors, resulting essentially in a clipping of the measurement, either on the low side of the intensity range or on the high side. The former case generally leads to a loss of resolution because of the natural decay of diffraction patterns towards high spatial frequencies, whereas the latter may compromise quantitativeness [40].

Despite these challenges, CDI has proved to work for many experimental environments mainly because of iterative algorithms that simulate the propagation and backpropagation numerically while carefully applying constraints in the real space, e.g. the support constraint, which eliminates many unknowns if the sample was isolated.

For ptychography, one scans a finite and coherent illumination, the probe *p*, across a sample (object *o*), collecting a diffraction image *I*(**v**;**y**) at each transversal shift **y** between the probe and object:

2.3 $$I(\text{v};\text{y})={\left|\int_{R^{2}}p(\text{x})o(\text{x}-\text{y})\cdot e^{-2\pi i\text{v}\text{x}}\;d\text{x}\right|}^{2}.$$

In conventional scanning transmission microscopy (STM), the image is formed by integrating the diffraction signal to form an image in real space: Image(y)=∫I(v,y) dv. This happens automatically when the detector is a single pixel, e.g. a diode. The final resolution in the image is limited by the size of the probe or (after deconvolution) by the step size *Δ***y**=|**y**_*j*+1_−**y**_*j*_| between adjacent scan points.

Ptychography combines STM with a pixelated detector and thus samples the diffraction signal *and* the position simultaneously. The large-angle diffraction signal holds interferometric information about the sample and probe on a grid which is finer than the scanning grid if the inverse of the maximum spatial frequency, **v**_max_, is smaller than the distance of the adjacent scan points *Δ***y**. As the resolution no longer directly depends on the step size, ptychography is often carried out with fewer samples but larger probes than STM and the continuous argument **y** is replaced by the scan point index *j*,

2.4 $$I_{j}(\text{v})={\left|\int_{R^{2}}p(\text{x})o(\text{x}-{\text{y}}_{j})\cdot e^{-2\pi i\text{v}\text{x}}\;d\text{x}\right|}^{2}=|F\{p(\text{x})o(\text{x}-{\text{y}}_{j})\}{|}^{2}.$$

Like single-shot CDI, ptychography uses an iterative approach to solve the phase problem. As mentioned in the Introduction, the key to the success of ptychography is the self-consistency of the sample transmission function for adjacent exposed areas. It reduces the number of independent variables encoded in the spatial frequencies of the diffraction pattern, yielding an overdetermined inverse problem—an effect similar to the support constraint of single-shot CDI, but without restricting the field of view. The redundancy induced by this overlap constraint has proved to be sufficiently robust for the illumination and object transmission to both be retrieved simultaneously. It also allows ptychography to be applied to a diverse set of more complicated models than (2.4) as we explain in the following section.

### (a) The models of ptychography

Important developments of ptychography hinge on the introduction of a new level of complexity compared with the model (2.4). Here, we highlight many of the important model updates and show how the model is incrementally adapted.

(i) *Propagation distance*: similar to single-shot CDI, ptychography uses a Fourier transform to alternate between two different domains and apply appropriate constraints. Like some imaging methods in the holographic regime, ptychography is not limited to far-field diffraction but may use any propagator that matches the experimental conditions. A refined model is written as
  2.5 $$I_{j}(\text{s})=|D_{\lambda ,z}\{p(\text{x})o(\text{x}-{\text{y}}_{j})\}{|}^{2},$$
  where $D_{\lambda ,z}$ is the operator that would propagate an electromagnetic wave from the source plane **x** to the detection plane **s** at a distance *z*. In the case of scalar diffraction theory, the angular spectrum representation is most general. It describes the propagation as a linear space-invariant filter [41]
  2.6 $$\psi (\text{s})=D_{\lambda ,z}\{\psi (\text{x})\}=\hat{F}\{F\{\psi (\text{x})\}\cdot \exp(2\pi iz\sqrt{\lambda^{-2}-{\text{v}}^{2}})\},$$
  where $H_{\lambda ,z}(\text{v})=\exp(2\pi iz\sqrt{\lambda^{-2}-{\text{v}}^{2}})$ is the associated transfer function for free-space propagation of a distance *z* for the wavelength λ. Applicability of such a modification was proven for imaging regimes of high Fresnel numbers [15,16].
(ii) *Sharing of diffraction data*: it is sometimes advantageous to combine lateral ptychographic scans as they share common information. Prominent examples are a common probe for a series of scans in tomographic ptychography [42] or a common object for measurements with and without beamstop or large segmented scans [43]. For each scan point index *j* that spans over multiple scans, the diffraction data may result from a set of different probe and object entities (*p*_*c*_ and *o*_*d*_). For example, the object index *d* may change, when the sample is rotated, and the probe index *c* may change because of optics or sample drifts. These changes may even occur within the same scan. We imply such changes with the assignments *c*=c(*j*) and *d*=d(*j*). Similarly, the geometry may otherwise vary between scans or scan points [31] and, consequently, we assume *z*=z(*j*). Hence, an improved model with respect to (2.5) can be written as
  2.7 $$I_{j}(\text{s})=|D_{\lambda ,z(j)}\{p_{c(j)}(\text{x})\cdot o_{d(j)}(\text{x}-{\text{y}}_{j})\}{|}^{2}.$$
  Except for ambiguous cases we will omit the scan point index *j* in the expressions d(*j*), c(*j*) and z(*j*).
(iii) *State mixtures*: it is rarely accurate to assume full coherence of the wavefield along the imaging pathway. Apart from the obvious partial coherence in the source, effects like signal spread in the detector or sample vibrations also yield the same common signature of reduced speckle or fringe visibility. It was shown recently [10] that ptychography can cope with all these experimental realities by introducing mixed states in the probe and/or object. This development has found immediate use and delivered high-quality reconstructions, e.g. for set-ups with a partially coherent source [10] (also in combination with signal spread in the detector [11]), or for rapid sample movement [12,44]. According to Thibault & Menzel [10], we extend the model in the following way:
  2.8 $$I_{j}(\text{s})=\sum_{m,n}|D_{\lambda ,z}\{p_{c,m}(\text{x})\cdot o_{d,n}(\text{x}-{\text{y}}_{j})\}{|}^{2}.$$
  The probe and object are composed of a finite number of coherent (pure) but mutually incoherent states. If the physical reality of the set-up does not deviate far from a pure state, the number of modes needed for convergence will be small. We have found that, in practice, allowing for a minimum of two or three modes in the probe always helps to reduce incoherence-related artefacts in the object [15].
(iv) *Polychromatic illumination*: traditionally, CDI experiments at synchrotron facilities are carried out with monochromatic sources which require a high degree of spectral filtering. For a single undulator harmonic of bandwidth *Δλ*/λ≈10^−2^, a common double crystal monochromator with bandwidth *Δλ*/λ≈10^−4^ filters roughly 99% of the available photons. While it has already been demonstrated that a monochromatic ptychographic model works almost unimpaired for bandwidths of up to 2% [11], higher bandwidths may require a model that takes the polychromaticity of the source into account:
  2.9 $$I_{j}(\text{s})=\int |D_{\lambda ,z}\{p(\lambda ,\text{x})\cdot o(\lambda ,\text{x}-{\text{y}}_{j})\}{|}^{2}\;d\lambda .$$
  Along with the obvious spectrum-dependent propagator, the object has a spectrally dependent response *o*(λ,**x**) and the source spectrum can be included in the probe *p*(λ,**x**). If the detector response is flat, equation (2.9) thus represents the polychromatic model where all spectral contributions form an integral. If there is a small sensitivity threshold for energy/wavelength or the source spectrum is comb-like, a discrete sum is also appropriate. In combination with equation (2.8), we arrive at
  2.10 $$I_{j}(\text{s})=\sum_{\lambda }\sum_{m,n}|D_{\lambda ,z}\{p_{c,m,\lambda }(\text{x})\cdot o_{d,n,\lambda }(\text{x}-{\text{y}}_{j})\}{|}^{2}.$$
(v) *Masking of diffraction data*: some pixels in the detector can yield bad data or no data at all if they are either over- or under-responsive, part of a module gap or even blocked by a central beam stop. Hence, a mask *M* is needed to disregard invalid pixels in the reconstruction algorithms. In general, this mask can be different for each diffraction pattern, for instance when pixels accidentally overexpose. The model thus becomes
  2.11 $$\begin{array}{rl} M_{j}(\text{s})I_{j}(\text{s}) & =M_{j}(\text{s})\sum_{m,n}\sum_{\lambda }|D_{\lambda ,z}\{p_{c,m,\lambda }(\text{x})\cdot o_{d,n,\lambda }(\text{x}-{\text{y}}_{j})\}{|}^{2} \end{array}$$
  2.12 $$\begin{array}{rl} & =M_{j}(\text{s})\sum_{\lambda ,m,n}|D_{\lambda ,z(j)}\{\psi_{j,\lambda ,m,n}(\text{x})\}{|}^{2}. \end{array}$$

In this last step, we have introduced the *elementary exit wave* *ψ*_*j*,λ,*m*,*n*_(**x**), which inherits all indices from *p* and *o*, and as such represents a coherent monochromatic wave after interacting with a single object mode.

## 3. A computational framework for ptychography

### (a) Design principles

It is apparent from (2.12) that developing reconstruction software for all models can be cumbersome. The contributions of various modes, probes, objects and propagators have to be accounted for in each algorithmic implementation by design or when an upgrade of existing code becomes necessary. Building a software from a simple model to more complex ones may lead to cluttered, unreadable code of monolithic structure with hidden switches, global parameters, etc. Such software is hard to maintain and debug and even harder to read for a novice.

To alleviate these programming difficulties—experienced first hand by the authors—we have decided to create PtyPy, an open-source ptychography software framework designed to achieve the following principles.

**Figure 1.**
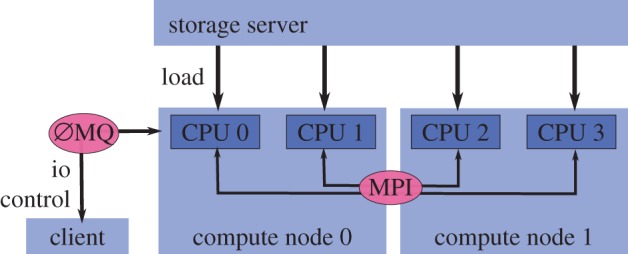
PtyPy relies on the *MPI* [50] protocol for parallel computation. Specifically, PtyPy distributes memory intensive data buffers, e.g. for the diffraction data and for the exit waves. Data can be loaded from the storage server in parallel if needed. The master node (here ‘CPU 0’) handles asynchronous communication with clients (e.g. for plotting), running a server based on *ZeroMQ* [51] in a shared-memory thread. (Online version in colour.)

(i) *Wide algorithm support*: while, in principle, any ptychographic algorithm is designed to reach a solution, the convergence properties of each algorithm can vary [45]. In practice, algorithms may converge at local minima (ePIE, ML) or explore the solution space without a clear stopping criterion (DM). A numerical implementation of a reconstruction algorithm together with a set of algorithm-specific parameters is called an *engine* in PtyPy. One can easily chain different engines sequentially to reach a specific objective, be it reaching the solution in the shortest computation time or pushing for the highest quality of the reconstruction.
(ii) *Wide model support*: a core design of PtyPy is the separation between the *models* and the *engines* in order to facilitate small and light-weight engine implementations that can be extended easily. As different experimental set-ups result in different models, PtyPy aims to support as many models as possible. Currently, PtyPy supports all models mentioned in the previous section and is ready to be adapted for new developments.
(iii) *Modularity and documentation*: providing a modular object-oriented implementation helps to extend and to reuse parts of it—a crucial feature if the code is understood as a continually developing project. For contributors, PtyPy is made of roughly 11 000 lines of Python code with an additional 7000 lines of comments or documentation strings, ensuring readability and a high degree of in-line documentation. For users, PtyPy is hosted online as an open-source project at http://ptycho.github.io/ptypy and provides tutorials and explanation along with documentation of all its classes and helper functions. In addition to core algorithmic functionality PtyPy features a rich set of utilities from plotting to parallel processing to common mathematical operations.
(iv) *Speed*: naturally, the ideal implementation finds the global solution in minimum time. For many algorithms, computation speed comes directly at the cost of flexible modular implementations. Fast implementations may require monolithic kernels to be built and compiled in a low level-language. This may imply fixing the ptychographic model and the engine algorithm to fit a specific problem while losing wide applicability in combination with an added effort to write the code. As a compromise, PtyPy relies on *NumPy* [46,47] for its calculations and thus benefits from optimized C-code for most numerically intensive operations. Accelerated engines can also be embedded in the same framework to keep the benefits of organization, storage and plotting purposes. A GPU-based DM engine has been successfully tested with PtyPy.
(v) *Wide end-user support*: preparing and organizing the data prior to reconstruction marks a major part of the implementation. The absence of standards means that the datasets are organized differently for each end-user application. Up to now, no data format for ptychographic datasets has been widely used, the hdf5-based [48] CXI-database [49] format being the closest current standard. PtyPy stores and loads data from hdf5 files with custom internal tree structures. It also provides an abstract base class for loading and preparation from raw datasets, which can be easily adopted to fit to a new application or data format.
(vi) *Scalability, interactivity and online capacity*: as for any other scanning method, ptychographic data are acquired as a stream. Depending on the instrument, the total acquisition time for a complete scan typically ranges from a few seconds to several minutes. Hence it may be useful for online feedback to initiate a reconstruction while data are streaming. The user should be able to oversee the reconstruction process in order to take immediate measures: abort the scan, adjust the sample position, etc. Although already achievable with current hardware, such dynamic ptychographic scans have not been used until now, mostly because of the complete absence of software to support these streams. PtyPy has been designed to provide such support: all internal storage containers can adapt to a continuous data inflow, and the reconstruction process is ready to start as soon as the first diffraction pattern is recorded. PtyPy is designed to run in parallel on many processing units, on a single computer or in a cluster. Larger data quantities may thus be analysed without compromise on processing time. The cluster of nodes running a reconstruction can be polled asynchronously and an interactive control of the reconstruction process following a client–server design is currently under development. Figure 1 gives a schematic of the implementation of distributed computing in PtyPy.

A detailed description of the implementation of all these principles is beyond the scope of this article but the reader is invited to browse the web page for documentation, or contact the authors for a copy of the software and to try one of the tutorials. Here, we concentrate on the core design of PtyPy, corresponding to elements (i) and (ii) above. For this purpose, PtyPy introduces a small set of connected Python objects which mediate between the physical world and its numerical representation, and organize the memory access to intuitively integrate algorithms as engines.

In the following, we describe PtyPy’s internal representation of the ptychographic model and the purpose of the main Python objects present in the framework. This description is illustrated with the implementation of a simple reconstruction engine.

### (b) Storage abstraction: the POD object

Any algorithmic implementation of ptychography starts with the problem of representing physical quantities as discrete memory buffers on the computer. The simplest solution is the use of array classes (such as the *numpy* nd-array [46]) that map physical quantities to multi-dimensional arrays, where each index represents one parametric dimension. For example, the array representing the probe may be five-dimensional with axes {*c*,*m*,λ,*x*_1_,*x*_2_}, where (*x*_1_,*x*_2_) is a suitable sampling of the spatial coordinate **x**. For the implementation of the model, the programmer has to find effective means to loop through these indices and to select or disregard the value of an index, depending on its physical meaning. One objective of PtyPy as a framework is to avoid such loops over memory buffer indices, as the same memory access pattern usually persists for all iterations of the engine. Instead, the engine is meant to loop over a single list of objects in which access rules for all memory buffers are stored.

**Figure 2.**
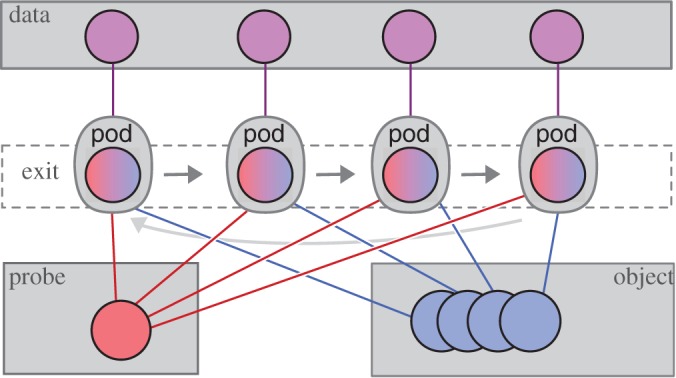
Schematic of PtyPy’s design principle. A ptychographic algorithm iterates over an assortment of objects called ‘pods’, which are used as an abstraction layer between engine algorithms and the access to diffraction, probe and object array access. (Online version in colour.)

**Figure 3.**
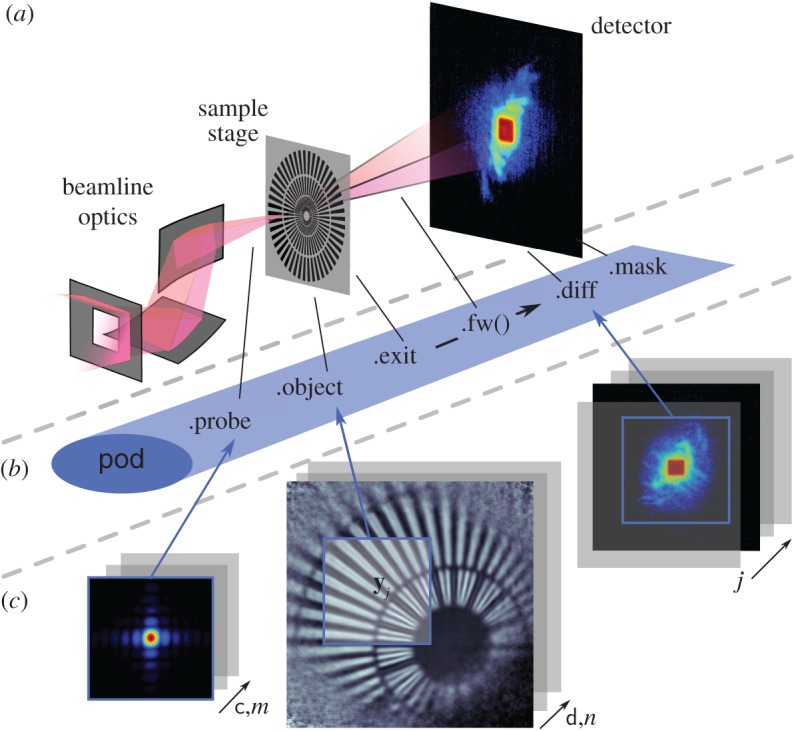
Main attributes of the pod object. (*a*) Elements of a typical diffraction set-up. (*b*) Corresponding attribute accesses of the Python object along the path of propagation. (*c*) Numerical arrays storing the data (*Storage* instances). *View* access to the data is indicated by blue squares of equal size.

The exit wave introduced in equation (2.12) can be seen to be the most elementary element to loop over as depicted in figure 2. In PtyPy, the information associated with a given exit wave is stored in a light-weight class called *Pod* (for **probe**-**object**-**diffraction**). Each *Pod* instance (hereafter simply referred to as pod) represents a unique combination of (*j*,λ,*m*,*n*), and thus stores access rules (e.g. memory addresses or slicing coefficients) to the corresponding memory buffers as described in table 1. In essence, a pod is equipped with everything to perform *one coherent propagation* of the ptychographic model as illustrated by figure 3. By design, a collection of pods alone suffices to calculate the forward model. The ‘Fourier modulus constraint’, imposed by the measured diffraction data, can thus be implemented using only pod instances as we can see in the following example.

**Table 1.** Attributes of a *Pod* instance (named pod) compared with their respective field representatives. In addition to the exit wave buffer pod.exit, a pod keeps attributes to access probe and object buffers through pod.probe and pod.object. It also carries access to diffraction data buffers (pod.diff) and the optional detector mask (pod.mask). The reference to the forward and the backward propagator is held in two methods, pod.fw() and pod.bw(), respectively.

pod attribute	field representative
pod.probe	*p*_c(*j*),*m*,λ_(**x**)
pod.object	*o*_d(*j*),*n*,λ_(**x**−**y**_*j*_)
pod.exit	*ψ*_*j*,λ,*m*,*n*_(**x**)
pod.diff	*I*_*j*_(**s**)
pod.mask	*M*_*j*_(**s**)
pod.fw	$D_{\lambda ,z(j)}$
pod.bw	${\hat{D}}_{\lambda ,z(j)}$

#### (i) Example (Fourier projection in a difference map)

According to Thibault *et al*. [26], the Fourier constraint for a single wave *ϕ* propagation to the far-field regime is given as

3.1 $$\phi^{\mathrm{it}+1}(\text{x})=\hat{F}\left\{F\left\{\phi^{\mathrm{it}}(\text{x})\right\}\frac{\sqrt{I(\text{v})}}{|F\left\{\phi^{\mathrm{it}}(\text{x})\right\}|}\right\}=\hat{F}\left\{F\phi^{\mathrm{it}}(\text{v})\cdot \Upsilon (\text{v})\right\},$$

where *Υ*(**v**) is the correction factor for the Fourier modulus to comply with the measured intensity data.

Within the difference map formalism with *β*=1 [52], the Fourier modulus constraint is not applied to the exit wave but rather to an expression involving the most recent updated probe, *p*^*it*^, and object, *o*^*it*^, and the exit wave, *ψ*^*it*−1^_*j*_, from the last iteration:

3.2 $$\phi_{j}^{\mathrm{it}}(\text{x})=2p^{\mathrm{it}}(\text{x})o^{\mathrm{it}}(\text{x}-{\text{y}}_{j})-\psi_{j}^{\mathrm{it}-1}(\text{x}).$$

The new set of exit waves is then

3.3 $$\psi_{j}^{\mathrm{it}+1}(\text{x})=\psi_{j}^{\mathrm{it}}(\text{x})+\phi_{j}^{\mathrm{it}+1}(\text{x})-p^{\mathrm{it}}(\text{x})o^{\mathrm{it}}(\text{x}-{\text{y}}_{j}).$$

For the most complex model (2.7), the input to the Fourier modulus constraint becomes

3.4 $$\phi_{j,m,n,\lambda }^{\mathrm{it}}(\text{x})=2p_{c,m,\lambda }^{\mathrm{it}}(\text{x})\cdot o_{d,n,\lambda }^{\mathrm{it}}(\text{x}-{\text{x}}_{j})-\psi_{j,m,n,\lambda }^{\mathrm{it}-1}(\text{x}).$$

Now, we can formulate the ‘Fourier update’ in a similar manner to that in [29] where we sum over all (coherent) exit waves that contribute to the same (partially coherent) signal in order to find the correction factor,

3.5 $$\Upsilon_{j}(\text{s})=1-M_{j}(\text{s})+\frac{M_{j}(\text{s})\sqrt{I_{j}(\text{s})}}{\sqrt{\sum_{\tilde{\lambda },\tilde{m},\tilde{n}}|D_{\lambda ,z}\{\phi_{j,\tilde{\lambda },\tilde{m},\tilde{n}}^{\mathrm{it}}(\text{x})\}{|}^{2}}}.$$

Finally, we replace the Fourier transform with an arbitrary propagator (2.6),

3.6 $$\phi_{j,m,n,\lambda }^{\mathrm{it}+1}(\text{x})={\hat{D}}_{\lambda ,z}\{D_{\lambda ,z}\{\phi_{j,m,n,\lambda }^{\mathrm{it}}(\text{x})\}\cdot \Upsilon (\text{s})\}$$

and

3.7 $$\psi_{j,m,n,\lambda }^{\mathrm{it}+1}(\text{x})=\psi_{j,m,n,\lambda }^{\mathrm{it}}(\text{x})+\phi_{j,m,n,\lambda }^{\mathrm{it}+1}(\text{x})-p_{j,m,\lambda }^{\mathrm{it}}(\text{x})\cdot o_{j,n,\lambda }^{\mathrm{it}}(\text{x}-{\text{x}}_{j}).$$

In the following, we show a possible implementation of the ‘Fourier’ modulus constraint using the *Pod* class of PtyPy. The function fourier_update takes as input a Python dictionary of pods that belong to the same scan point index (*j*). All these pods represent the mixed states and spectral compositions of the experiment. The associated exit waves *ψ*^*it*^_*j*,λ,*m*,*n*_ are updated simultaneously as the feedback factor *Υ*(**s**) from the Fourier update is the same for one diffraction pattern.


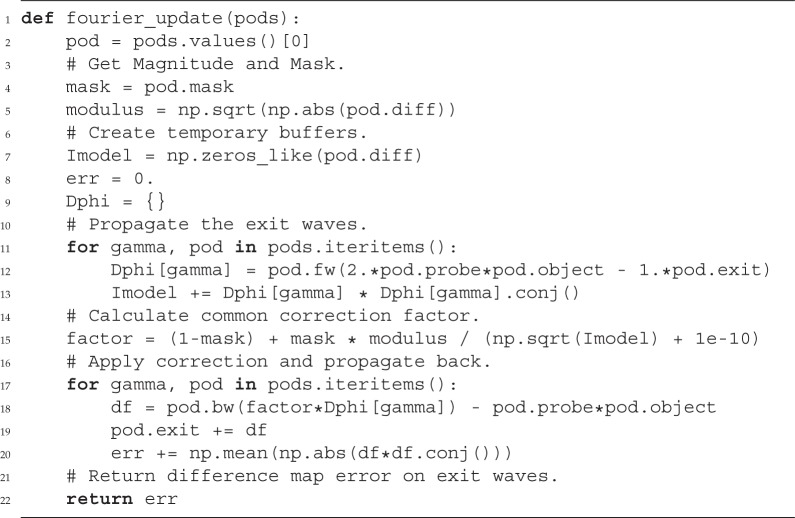


### (c) Data constructs and access

**Figure 4.**
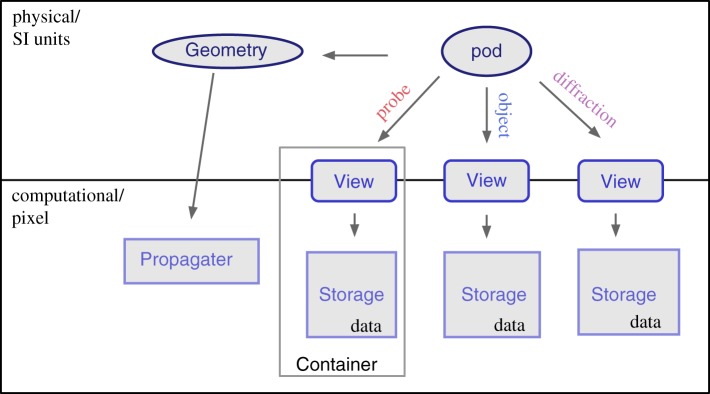
Overview of the most important classes in PtyPy and how they relate to each other. The *Pod* class is the highest level object. In order to be able to create the forward model, it contains a (data)–*View* instance for each entity (three are shown) and a reference to the scan’s geometry in order to use the appropriate propagator. The *View* class mediates between ‘physical’ wavefields and ‘numerical’ data buffers, and, when applied to a *Storage* instance, it yields the two-dimensional data representing the region of interest of the view. The *Container* class instance manages views and storages and there is one *Container* instantiated for each entity. The *Geometry* class contains the scan geometry, i.e. distance from the detector to object and pixel size, resolution, etc. It provides the numerical propagator for coherent forward and backward propagation. (Online version in colour.)

The *Pod* class is convenient when accessing data from the buffer arrays but these arrays also need to be created, managed and contained by other objects. PtyPy provides three other basic classes for data storage and access. For a compact overview, see figure 4.

View In numerical computations, we cannot work with infinitely extended wavefields, but rather with compact rectangular regions. A *View* instance (hereafter view) stands for such a region and is characterized by its extent and the physical position of its centre relative to the wavefields coordinate origin. A pod therefore comprises a view for each of the five entities probe, object, exit wave, diffraction data and mask. The number of views scales with the number of scan points.
Storage A *Storage* instance (hereafter storage) is a numerical array associated with a physical coordinate system. It represents a set of wavefields that share the same coordinate system. It can adapt its internal array size dynamically depending on those parts of the wave field that are requested by views. The number of storages scales roughly with the number of scans that are being organized by PtyPy. Applying a view to a storage yields the two-dimensional array which is sliced from the storage’s data array and represents the numerical data for the view’s physical extent:
  storage[view] = data (2d)
Container A *Container* instance (hereafter container) holds all views and storages that belong to the same entity in ptychography. In contrast to the *View* class and the *Storage* class, there are only five base *Container* instances in PtyPy, one for each entity. The number of storages depends on the number of scans and their sharing behaviour. Applying a view to its container is the same as applying the view to its associated storage in the container:
  container[view] = data (2d).

A container is also capable of creating copies of itself (clones) or performing basic mathematical operations which it will relay to all its storages’ internal data buffers. The ability to clone is important for algorithms to provide temporary data buffers that behave like one of the entities. In addition to basic in-place maths operations, the container is also capable of communicating its data among processes in the case where the same PtyPy reconstruction runs in parallel on many nodes (figure 1).

#### (i) Example (overlap projection in a difference map)

In its simplest form [26], the probe and object update can be written as

3.8 $$o^{\mathrm{it}+1}(\text{x})=\frac{\sum_{j}{\left[p^{\mathrm{it}}(\text{x}+{\text{y}}_{j})\right]}^{*}\cdot \psi^{\mathrm{it}}(\text{x}+{\text{y}}_{j})}{\sum_{j}|p^{\mathrm{it}}(\text{x}+{\text{y}}_{j}){|}^{2}}$$

and

3.9 $$p^{\mathrm{it}+1}(\text{x})=\frac{\sum_{j}{\left[o^{\mathrm{it}}(\text{x}-{\text{y}}_{j})\right]}^{*}\cdot \psi^{\mathrm{it}}(\text{x})}{\sum_{j}|o^{\mathrm{it}}(\text{x}-{\text{y}}_{j}){|}^{2}}.$$

If we include the model mentioned in §2a, we have to sum also over shared data and other modes of the probe and object. For one of the objects *o*_*d*_, we have to restrict the update to those scan point indices *i* that contribute to that object, i.e. *i*∈{*j* | d(*j*)=*d*}:

3.10 $$o_{d,n,\lambda }^{\mathrm{it}+1}(\text{x})=\frac{\sum_{i,m}{\left[p_{c(i),m,\lambda }^{\mathrm{it}}(\text{x}+{\text{y}}_{i})\right]}^{*}\cdot \psi_{i,m,n,\lambda }^{\mathrm{it}}(\text{x}+{\text{y}}_{i})}{\sum_{i,m}|p_{c(i),m,\lambda }^{\mathrm{it}}(\text{x}+{\text{y}}_{i}){|}^{2}}.$$

The same holds true for the probes. For probe *c*, we pick the indices *i*∈{*j* | c(*j*)=*c*} and arrive at a similar expression:

3.11 $$p_{c,m,\lambda }^{\mathrm{it}+1}(\text{x})=\frac{\sum_{i,n}{\left[o_{d(i),n,\lambda }^{\mathrm{it}}(\text{x}-{\text{y}}_{i})\right]}^{*}\cdot \psi_{i,m,n,\lambda }^{\mathrm{it}}(\text{x})}{\sum_{i,n}|o_{d(i),n,\lambda }^{\mathrm{it}}(\text{x}-{\text{y}}_{i}){|}^{2}}.$$

Other than the Fourier update (p. 11), the probe and object update require a copy of the same kind as the probe and object in order to calculate the denominator in the equations above. Despite the complexity in (3.10) and (3.11), the algorithmic implementation in PtyPy resembles very much (3.8) and (3.9) regarding its simplicity.


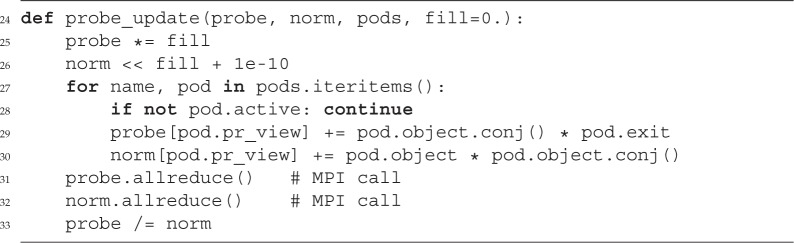


In addition to the probe container, the update functions require a container of similar shape which is called norm here. Access to the data of that container happens through the probe-view as norm was initially a copy of probe. As announced before, we note the in-place operation ‘*=’, ‘<<’ and ‘/=’ in lines 25, 26 and 33, respectively, and the parallel reduction calls in lines 31 and 32 which perform a sum (if no argument is given).

The object update is of a similar kind and benefits in its simplicity additionally from the implicit shift of the object views.


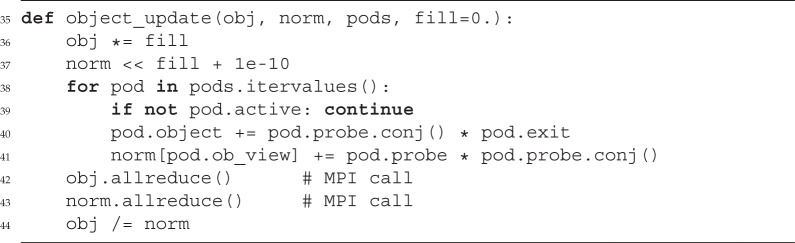


We observe that, although the model can be very complex, the algorithmic core stays compact and simple. There is no ‘boilerplate code’ from nested loops and no indexing of memory buffers. The functions are also compatible to parallel execution with MPI.

### (d) A cross-referenced network

All instances of the basic classes defined in PtyPy are accessed through multiple cross-references. The highest-level entry point is the object ptycho, which gives direct access to all pods (ptycho.pods), the five *Container* instances for the five entities (ptycho.probe, ptycho.obj, etc.) and the scan geometries. Other objects are accessed through a hierarchy of attributes. For instance, ptycho.diff.views provides access to all diffraction data frames, while view.owner refers to the container that holds the view.

#### (i) Example (a simple difference map algorithm)

Provided with a ptycho instance and the functions mentioned before, we are able to write a simple reconstruction engine.


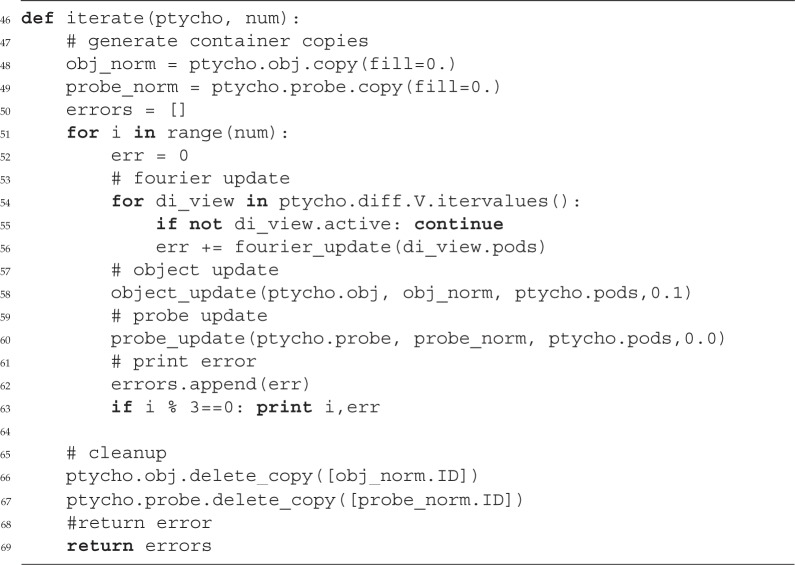


Certainly, the example engine iterate requires a compatibly-built Python object instance ptycho to actually work. Depending on an extensive input parameter tree, PtyPy provides a ptycho instance along with the pods and initialized storages for the probe and object. More information on this initialization procedure can be found in the source code or the tutorials provided in the electronic supplementary material.

## 4. Examples

### (a) Visible light ptychography of a standard resolution target

**Figure 5.**
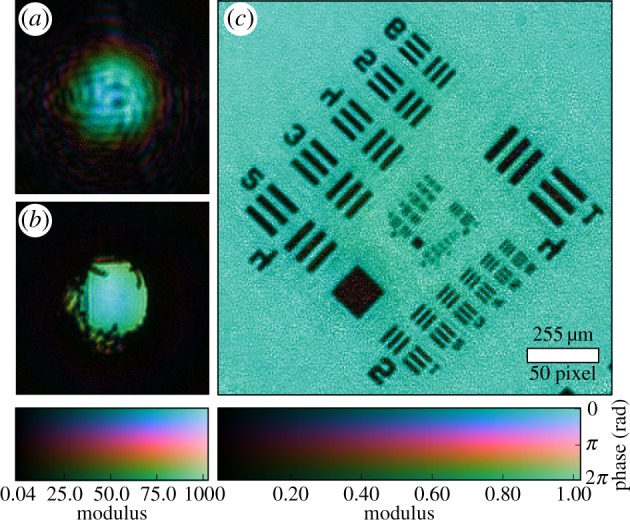
Ptychography experiment with visible light in a laboratory. Complex values are colour-coded such that the phase maps to hue and the modulus maps to the luminance of the image. (*a*) Recovered complex wavefront of the illumination in the sample showing a high degree of diversity. (*b*) Illumination in the aperture plane was obtained from a numerical propagation of (*a*). (*c*) Image of the recovered object transmission function of the sample. The noise in the reconstruction is caused by a low signal-to-noise ratio at high angular frequencies, which, in turn, is a result of the isotropic readout noise of the CCD.

As a first experimental demonstration of the package, we present a reconstruction from a simple laser diffraction experiment using a set-up similar to one previously reported [53]. In this experiment, an aperture and a sample are placed along the optical path between an LED laser (650 nm wavelength) and a CCD. The aperture, a hole pierced in a piece of cardboard with a fine needle, produces at the sample plane 11.5 mm downstream an illumination with high angular diversity, as shown by its reconstruction in figure 5*a*. The back-propagated illumination at the aperture plane is shown in figure 5*b* and a diameter of roughly 300 μm can be observed.

The detector, a Fingerlakes P1001 monochrome CCD camera, is placed 145 mm behind the sample plane. An additional neutral density filter upstream of the camera is used to match the laser intensity with the minimal CCD exposure time to avoid overexposure. The diffraction images are binned by 3×3, creating effective detector pixels of 72×72 μm area. In total, 201 diffraction images were acquired on a non-periodic grid in a square area of 2 mm side length. The resolution of roughly 100 mm^−1^ and a total photon count of roughly 2.38×10^9^ results in about 10^4^ photons per reconstruction pixel. After acquisition, the diffraction data were processed into PtyPy’s hdf data format as described in the electronic supplementary material (http://ptycho.github.io/ptypy/rst/data_management.html#ptyd-file).

The ptychographic reconstruction of a USAF optical resolution target is shown in figure 5*c*. For this reconstruction, we used the code snippets presented in the previous section of this manuscript. The remaining part of the reconstruction script is given below.


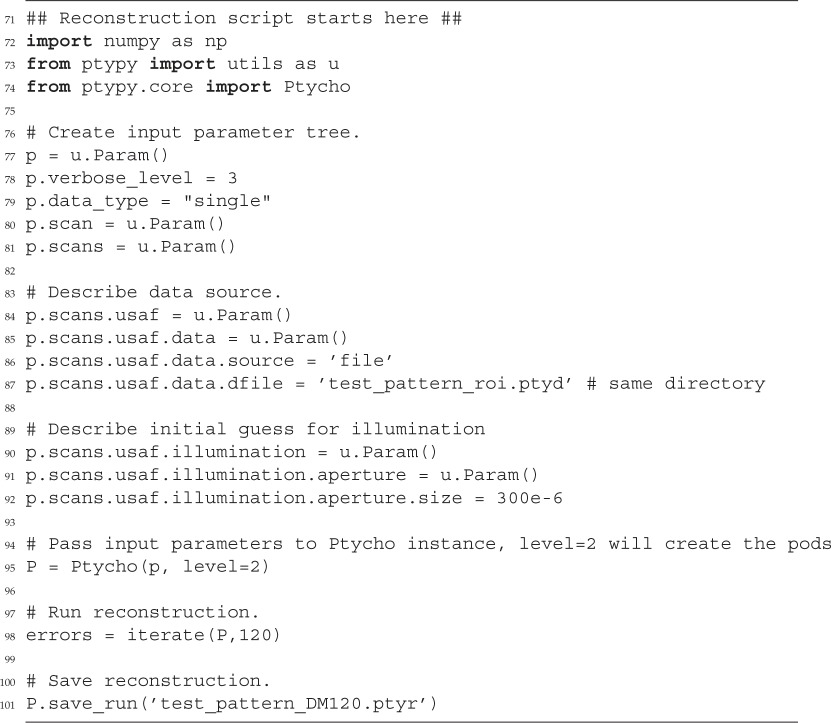


The noise visible in the object reconstruction originates from the absence of any corrective measures in the algorithm such as a Fourier relaxation threshold [54] or gradient regularization [29]. However, noise correction was left out on purpose to not misdirect the reader from the core principles by filling the presented code with non-essential instructions. The full capabilities of the mature reconstruction engines are demonstrated in the next section.

### (b) X-ray ptychography of a zone plate at a synchrotron facility

A ptychographic dataset of a zone plate was acquired on a rectangular raster grid pattern with 100 nm step size at a photon energy of 6.8 keV. The probe was a cone beam formed from a pinhole aperture in front of a focusing zone plate optic. A photon counting detector of 172 μm pixel size recorded diffraction images 2.19 m downstream behind the zone plate specimen. These data were originally published in [7]. We refer to that publication for more details.

Here, a 7×7 μm region of the original data is reconstructed with PtyPy using 300 iterations of DM with a subsequent ML refinement of 300 iterations. Five probe modes are allowed to account for possible decoherence effects.

**Figure 6.**
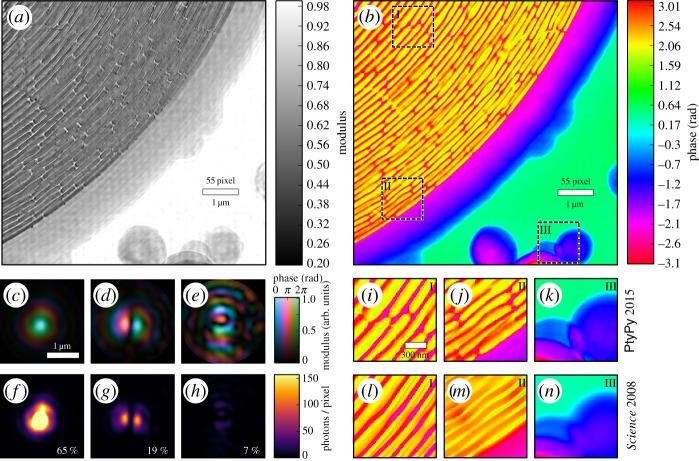
X-ray ptychography at a small-angle scattering beamline of a synchrotron. (*a*,*b*) Recovered modulus and phase of the outer region of a zone plate, serving here as a sample. (*c*–*e*) Wavefields of three recovered probe modes that represent 91% of the illumination in the sample plane. The probe modes were orthogonalized with the main mode being the mode to the left (*c*). (*f*–*h*) The same probe modes but their intensity distribution is displayed on a linear scale. Note that the main mode reaches values up to 700 photons in its centre. The relative power of the mode is shown in the bottom right corner. (*i*–*k*) Selected regions of the recovered phase (*b*) displayed alongside the same regions (*l*–*n*) from the original phase reconstruction [7] of the sample. An improvement in reconstruction quality is apparent: small bridges for stabilizing the ring structure are visible in (*i*) but not in (*l*). Deviations of the outer zones from a circular path can be observed in (*j*) but can only be guessed from (*m*).

We notice that the object transmission can be recovered in fine detail as shown by figure 6*a*,*b*. More specifically, the reconstruction quality is higher than the original as emphasized by panels (*i*)–(*n*), which compare selected regions with the original and the new reconstruction. It is a surprising result as only 25% (i.e. 35×35 of 70×70 scan points) of the available diffraction patterns are used in this reconstruction. One reason that may have prevented the original algorithm from resolving the specimen with fine detail can be found in the mode decomposition (figure 6*c*–*e*) of the probe: a significant amount of energy (35%) is accumulated in secondary modes indicating that the illumination onto the pinhole was not entirely coherent.

## 5. Conclusion

In this paper, we have shown that many different models of ptychography may be unified into a single model. We showed how a small set of abstract Python classes can be used to capture this model in a cross-referencing network. The references (API) exposed by the network were used to demonstrate how algorithms may be written in a way that is agnostic of the underlying physical geometry—an important trait for decoupling the algorithmic implementation from experimental specifications. We showed that many of today’s applications are included in the formalism presented here, and it is our opinion that many future problems of ptychography may be rephrased in a similar manner to extend the scope of our framework.

PtyPy currently provides parallel computation of distributed data, an abstract loading class to accommodate different instruments and storage environments, and a defined structure for raw and reconstructed data. As a framework, it exposes all its functions and classes to the user for free adaptation and provides a rich set of utility functions of practical use for many aspects of ptychography. The future roadmap includes GPU accelerated algorithms, on-the-fly adaptation and control of engine parameters as well as additional plug-ins for beamlines or other instruments that wish to use PtyPy for ptychographic reconstructions.

## Supplementary Material

Example ptychographic dataset (Figure 5)

## Supplementary Material

Example reconstruction script

## Data accessibility

The dataset of the visible light ptychography experiment and an example Python script for reconstruction have been uploaded as part of the electronic supplementary material. The software, PtyPy, is available for academic research in the X-ray regime and for numerical simulations. It can be obtained through personal enquiry to the authors. The documentation for all functions and parameters, as well as tutorials and further details about data management, are available on http://ptycho.github.io/ptypy.
